# Case Report: Tibial plateau fracture in Charcot neuroarthropathy: a report of two cases with contrasting outcomes

**DOI:** 10.3389/fsurg.2026.1840567

**Published:** 2026-05-28

**Authors:** Weikun Li, Yan Zhang, Hui Wang

**Affiliations:** Department of Knee Joint, Mianyang Orthopaedic Hospital, Mianyang, Sichuan, China

**Keywords:** Charcot neuroarthropathy, diabetes mellitus, misdiagnosis, tibial plateau fracture, total knee arthroplasty

## Abstract

Charcot neuroarthropathy (CNA) is a debilitating complication of diabetes mellitus that often poses diagnostic challenges, particularly when associated with fractures around the knee joint. This case series describes two contrasting cases of CNA manifesting as tibial plateau fractures in patients with type 2 diabetes. In the first case, a 55-year-old woman with poorly controlled diabetes sustained a tibial plateau fracture following a minor sprain. The fracture was misdiagnosed as an old comminuted tibial plateau fracture, and the patient underwent open reduction and internal fixation (ORIF) with iliac bone grafting. Intraoperative findings revealed severe bone fragmentation, osteolysis, and osteoporosis—features suggestive of underlying CNA, which were not recognized at the time. The surgery ultimately failed because of implant displacement and osteochondral collapse. In the second case, a 65-year-old man with well-controlled diabetes presented with acute knee pain and swelling after minimal trauma. Early diagnosis of CNA (modified Eichenholtz stage I) was established, and following a period of conservative management, the patient underwent total knee arthroplasty with stemmed components. At 9-year follow-up, the patient remained free of complications with satisfactory joint function. These cases highlight the critical importance of maintaining a high index of suspicion for CNA in diabetic patients presenting with fractures that are disproportionate to the mechanism of injury. Early recognition is essential to avoid inappropriate surgical interventions, and when joint destruction is advanced, total knee arthroplasty can yield durable long-term outcomes.

## Introduction

Charcot neuroarthropathy (CNA) is one of the most devastating musculoskeletal complications of diabetes mellitus, yet it remains frequently underrecognized in clinical practice (1). Originally described by Jean-Martin Charcot in 1868 in association with tabes dorsalis, this progressive, non-infectious destructive arthropathy is now most commonly encountered in patients with long-standing diabetes and peripheral neuropathy (2). The condition is characterized by an initial inflammatory phase, followed by progressive joint destruction, fragmentation, and ultimately severe deformity with functional impairment (2).

The pathophysiology of CNA is complex and incompletely understood, involving a combination of autonomic dysregulation leading to hyperemia and osteopenia, repetitive microtrauma in the setting of protective sensory loss, and aberrant inflammatory responses that perpetuate a cycle of destruction and failed repair (3). The resulting bone fragility predisposes affected patients to pathological fractures and dislocations, often following minor or even unrecognized trauma. While the foot and ankle are the most frequently involved sites, CNA of the knee joint—so-called “Charcot knee”—presents unique diagnostic and therapeutic challenges because of the weight-bearing demands of the joint and the complex biomechanics involved (4).

The diagnosis of CNA is notoriously difficult, particularly in its early stages. Clinical presentation may be subtle, with patients often reporting only mild discomfort or swelling despite advanced radiographic destruction—a phenomenon attributable to concomitant sensory neuropathy. This clinical–radiographic dissociation frequently leads to delayed or incorrect diagnoses, with CNA being mistaken for infectious, neoplastic, or degenerative conditions (5). The consequences of misdiagnosis can be catastrophic: inappropriate surgical interventions, such as open reduction and internal fixation of what is mistakenly perceived as an isolated traumatic fracture, are associated with exceptionally high failure rates because of poor bone quality, impaired healing capacity, and the absence of protective sensation, which can lead to unprotected weight-bearing on compromised constructs (6).

Tibial plateau fractures occurring in the setting of underlying CNA represent a particularly treacherous clinical scenario. The radiographic appearance of a comminuted, seemingly traumatic fracture may overshadow the subtle clues suggestive of an underlying neuropathic process, especially in patients without a previously established diagnosis of CNA. The literature on this specific association remains sparse, and clinical guidance for management is limited. Conversely, when CNA is recognized early, appropriate interventions—including protected weight-bearing, pharmacological modulation of bone resorption, and ultimately joint arthroplasty with specialized techniques—can yield favorable long-term outcomes (7, 8).

Herein, we present two contrasting cases of CNA involving tibial plateau fractures in patients with type 2 diabetes mellitus. The first case illustrates the devastating consequences of misdiagnosis and inappropriate surgical intervention, while the second demonstrates the favorable outcome achievable with timely recognition and staged management culminating in total knee arthroplasty (TKA). These cases aim to increase clinical awareness of this underrecognized entity and provide practical insights into its diagnosis and management.

## Case report

Chronological summary of the two contrasted cases is presented in Table 1.

**Table 1:** Chronological summary of the two contrasted cases.

Time point	Case 1 (misdiagnosis pathway)	Case 2 (early recognition pathway)
Baseline	1-year history of diabetes mellitus (DM), self-stopped medications 4 months prior	10-year history of DM, stable insulin
Day 0	Minor sprain, weight-bearing	Pop while pouring water, ambulatory
Week 2–3	X-ray/MRI: comminuted fracture, referred	–
Day 30	Admitted; HbA1c 14.2%	–
Day 34	ORIF + bone graft (29 days postinjury)	–
Month 2	–	MRI: CNA stage I; HbA1c 6.9%; strict non-weight-bearing (NWB) + bisphosphonate
Month 4	–	Radiographs show progression; TKA offered
Month 7	–	TKA with stemmed components
Month 11	Implant failure, revision declined	–
Year 1	–	range of motion (ROM) 0°–110°, pain-free
Year 9	–	Implant stable, no complications

### Case 1

A 55-year-old Asian woman with a 1-year history of type 2 diabetes mellitus presented to our hospital with right knee pain and limping. The clinical timeline was reconstructed as follows. Four months prior to presentation, she had self-discontinued all oral hypoglycemic agents. One month before admission (day 0), she sustained a minor sprain while walking in a park and immediately developed pain; however, she remained able to bear weight and ambulate with discomfort. Approximately 2–3 weeks later (days 14–21), initial radiographs and magnetic resonance imaging (MRI) performed at a local hospital revealed a comminuted fracture of the medial tibial plateau with articular surface fragmentation and collapse. On day 30, she was admitted to our center, where she continued to walk with partial weight-bearing and a limp.

Neuropathy assessment on admission revealed decreased vibration sensation (tuning fork 128 Hz: 4/8 at the first metatarsophalangeal joint bilaterally) and absent ankle reflexes. Semmes–Weinstein 10-g monofilament testing was positive for loss of protective sensation in both feet. No formal nerve conduction studies were performed at this stage. Physical examination revealed a varus deformity of the right knee with moderate swelling. Local skin temperature was slightly elevated, but tenderness was minimal despite the extent of radiographic findings. Range of motion was limited to 5°–100° (extension to flexion). Laboratory evaluation on admission (day 30) confirmed critically poor glycaemic control, with a fasting blood glucose level of 18.32 mmol/L and glycated hemoglobin (HbA1c) of 14.2%. Key baseline and exclusion parameters were as follows: white blood cell count 7.8 × 10⁹/L (normal 3.5–9.5) with 68% neutrophils; C-reactive protein (CRP) 15.4 mg/L (normal < 8); erythrocyte sedimentation rate (ESR) 38 mm/h; alkaline phosphatase 145 U/L; and serum albumin 32 g/L. Renal and hepatic function tests were within normal limits. Serologic screening and tumor markers were unremarkable. Bone densitometry confirmed severe osteoporosis, which likely contributed to the severity of fragmentation seen on subsequent imaging. Computed tomography (CT) imaging showed a comminuted fracture of the medial tibial plateau with significant bone fragmentation and defects (Figure 1).

**Figure 1:**
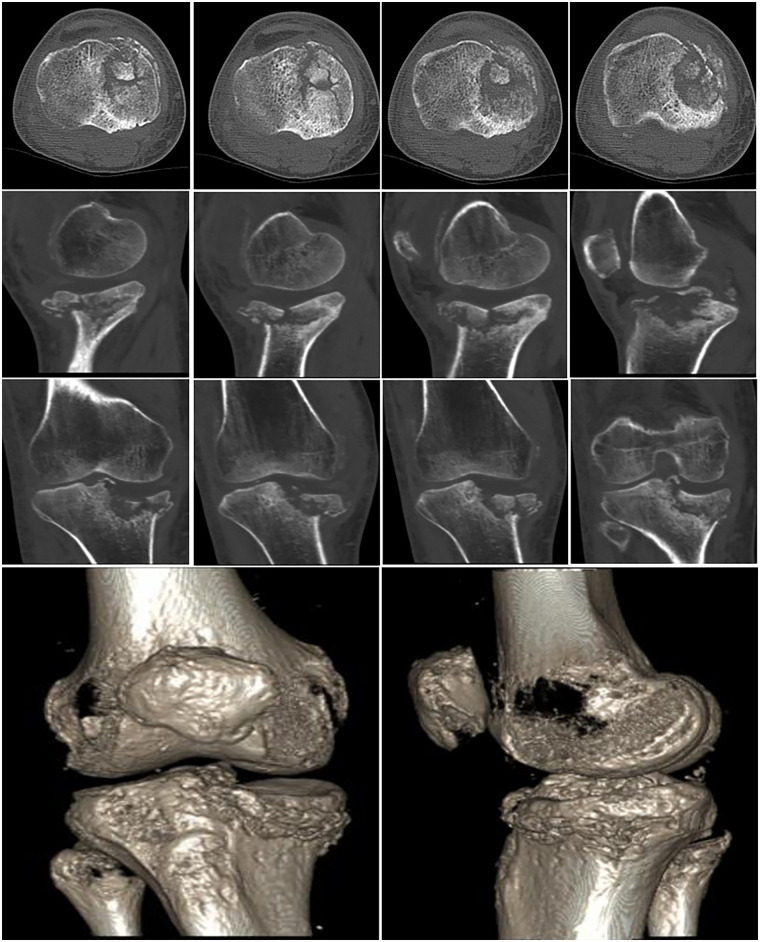
CT imaging of the right knee (coronal and sagittal reconstructions). A comminuted fracture of the medial tibial plateau is seen with significant bone fragmentation and a large subchondral defect. The lateral compartment and patellofemoral joint were preserved.

The patient was initially diagnosed with an old right tibial plateau fracture (Schatzker type IV) in the setting of uncontrolled diabetes mellitus and severe osteoporosis. Following 4 days of endocrine consultation for glycaemic optimization, on day 34 (29 days postinjury), she underwent open reduction and internal fixation with autologous iliac bone grafting via an anteromedial approach. Intraoperatively, pale yellow effusion was noted, and the medial tibial plateau was found to be severely comminuted with osteolysis, multiple free bone fragments, and pronounced local osteoporosis—findings suggestive of an underlying neuropathic process. Intraoperative synovial fluid analysis revealed a pauci-cellular aspirate (white cell count 1,200 cells/µL, predominantly mononuclear), while Gram staining, culture, and crystal analysis were negative. Pathological examination of bone tissue revealed focal callus formation without evidence of infection or neoplasm (Figure 2). Postoperative follow-up imaging within several days demonstrated satisfactory internal fixation (Figure 3), and the patient was instructed to maintain strict non-weight-bearing status for 3 months.

**Figure 2:**
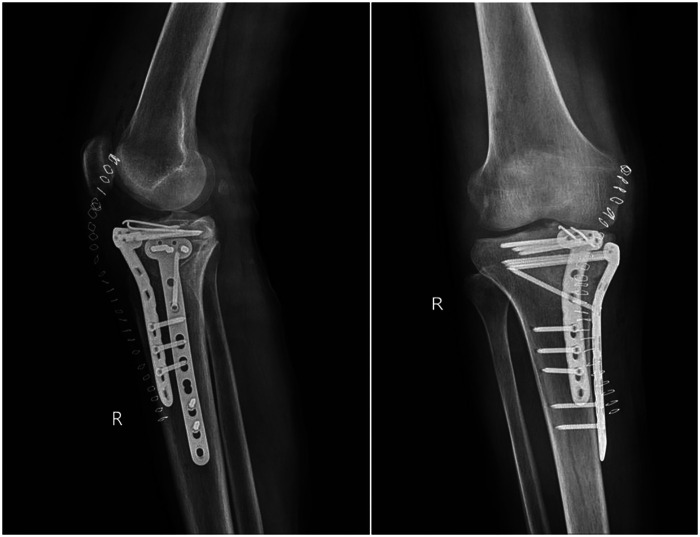
Pathological examination of bone tissue revealing focal callus formation.

**Figure 3:**
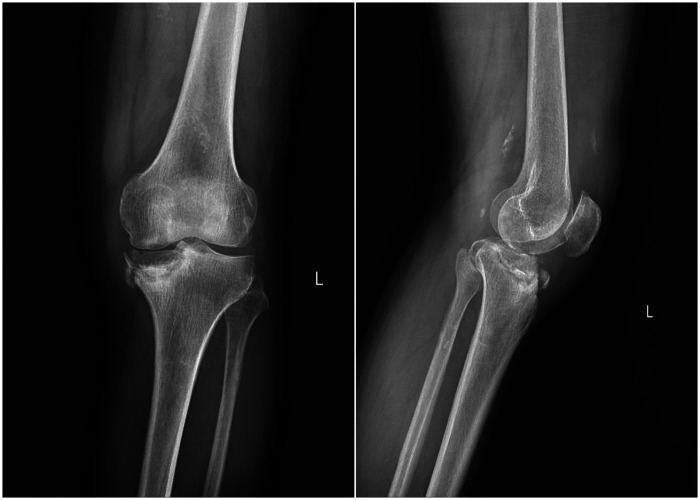
Postoperative radiographic evaluation demonstrating satisfactory placement of the internal fixation device.

The patient subsequently failed to attend all scheduled follow-up visits at 1, 2, 3, and 6 months postoperatively. Ten months after surgery (month 11 of the overall timeline), she returned with recurrent knee pain and claudication. Radiographs demonstrated failure of internal fixation, with displacement of Kirschner wires and collapse of the medial tibial plateau (Figure 4). Revision surgery was recommended, but the patient declined further intervention.

**Figure 4:**
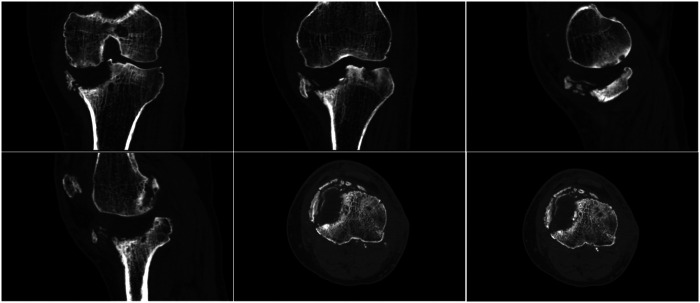
Radiographic examination at 10 months postoperatively revealing failure of internal fixation, manifested as Kirschner wire displacement and collapse of the medial tibial plateau.

### Case 2

A 65-year-old Asian man with a 10-year history of type 2 diabetes mellitus, well controlled on a stable insulin regimen, presented to the outpatient clinic with acute left knee pain and swelling. The clinical timeline was documented as follows. On day 0, the patient reported hearing a popping sound in the knee while pouring water and subsequently experienced pain, although he remained ambulatory. Physical examination revealed mild swelling and tenderness localized to the medial joint space with a full range of motion. Distal neurovascular examination was unremarkable. Initial radiographs showed no obvious abnormalities (Figure 5). Conservative treatment with analgesics was prescribed.

**Figure 5:**
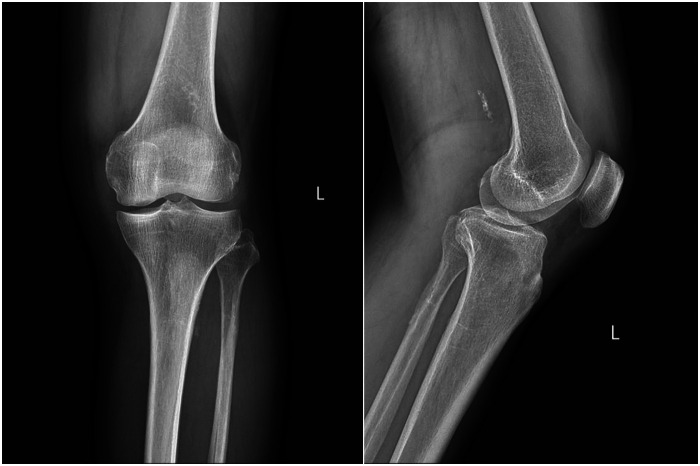
Initial radiographs of the left knee showing no obvious fracture findings.

Two months later (month 2), the patient returned because of persistent pain. MRI of the left knee demonstrated a comminuted and collapsed fracture of the medial tibial plateau with partial bone resorption, extensive bone marrow edema involving both the femoral condyle and tibial plateau, joint effusion, and periarticular soft tissue swelling (Figure 6). Neurovascular evaluation confirmed distal symmetric polyneuropathy: vibration perception threshold was absent at the hallux, the 5.07 Semmes–Weinstein monofilament was not felt at three of four plantar sites on each foot, and Achilles reflexes were absent. Nerve conduction velocity of the sural nerve could not be obtained bilaterally. At this stage, laboratory parameters confirmed his well-controlled metabolic status and revealed an acute sterile inflammatory response: average daily blood glucose during the preceding week ranged from 5.8 to 8.2 mmol/L, HbA1c was 6.9%, CRP was 10.5 mg/L, ESR was 22 mm/h, and complete blood count and differential were within normal limits. Serum C-terminal telopeptide of type I collagen (CTX-1) was elevated at 0.850 ng/mL (reference 0.100–0.700), indicating increased bone resorption. Renal function was normal (estimated Glomerular Filtration Rate (eGFR) 82 mL/min/1.73 m^2^). Infection was systematically excluded: serial measurements of CRP and ESR showed no upward trend, the knee exhibited no erythema or skin ulceration, and aspiration was not performed because the clinical presentation was not suggestive of infection. Based on the combination of minimal trauma, confirmed diabetic neuropathy, imaging findings, and the classic clinical–radiographic dissociation (extensive joint destruction with relatively preserved range of motion and modest pain), a diagnosis of CNA (modified Eichenholtz stage I) was established.

**Figure 6:**
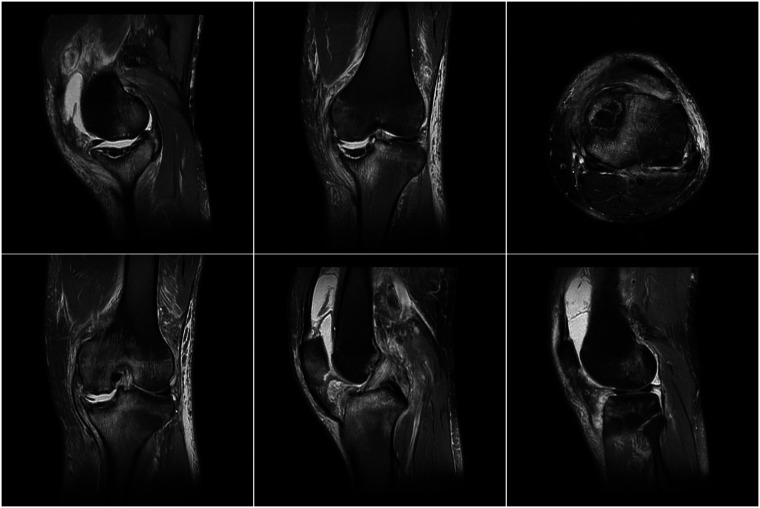
MRI findings of the left knee demonstrating a comminuted and collapsed fracture of the medial tibial plateau with evidence of partial bone resorption, accompanied by extensive bone marrow edema involving the femoral condyle and tibial plateau. Additional positive findings included joint effusion, thickening of the medial joint capsule, and periarticular soft tissue swelling.

The patient was immediately managed with a custom-molded double-upright unloader knee brace locked in extension during ambulation, with strict non-weight-bearing maintained for a total of 2 months. In addition, bisphosphonate therapy (oral alendronate 70 mg once weekly) was initiated because of the markedly elevated CTX-1 level and the concept that osteoclast-mediated bone resorption is a hallmark of the acute phase of Charcot neuroarthropathy. Follow-up radiographs obtained 2 months later (month 4) showed progressive changes (Figure 7). Total knee arthroplasty was recommended; however, the patient initially deferred surgical intervention. Three months later (month 7 from injury, month 5 from diagnosis), he returned, and CT imaging confirmed rapid progression of joint destruction (Figure 8). A cemented total knee arthroplasty was subsequently performed using a constrained condylar prosthesis with 100-mm cemented femoral and 120-mm cemented tibial stem extensions (diameter 14 mm; both press-fit distally) to bypass the metaphyseal bone defects. Intraoperatively, the medial tibial plateau was found to be completely collapsed with extensive bone loss; the defect was managed using a 10-mm medial tibial augment combined with cement filling (Figure 9a). Ligament balancing required release of the superficial medial collateral ligament, and a constrained insert was selected because of moderate mediolateral laxity after gap balancing. A deep drain was placed and removed 24 h postoperatively.

**Figure 7:**
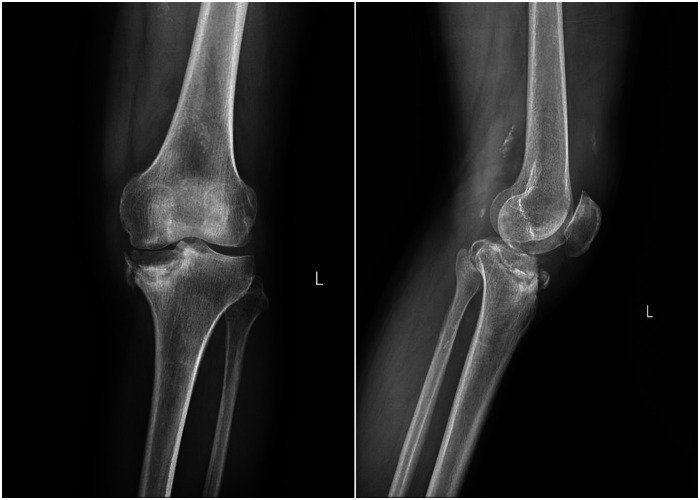
Radiographs of the left knee revealing a comminuted and collapsed fracture of the medial tibial plateau with bone resorption and destruction.

**Figure 8:**
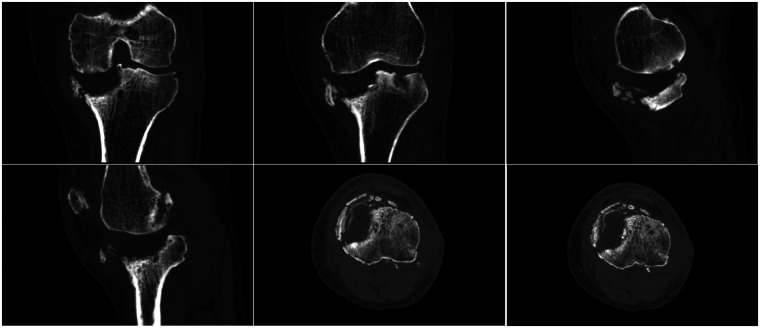
Compared with previous imaging, the CT scan of the left knee revealed rapid and progressive local bone destruction.

**Figure 9:**
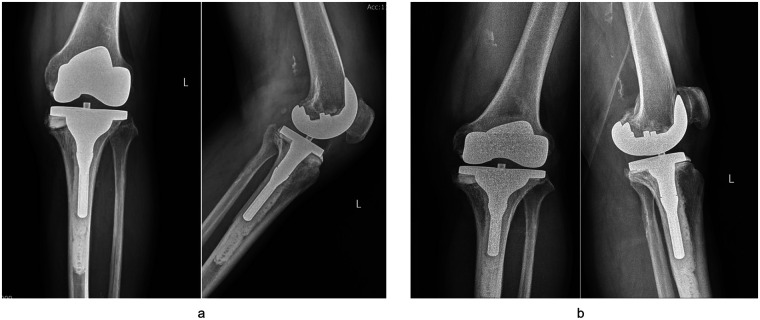
**(a)** Postoperative radiographs following cemented and stemmed total knee arthroplasty of the left knee. **(b)** Follow-up radiographs at 9 years demonstrating satisfactory prosthesis fixation.

Postoperatively, the patient followed a structured, protected weight-bearing protocol for 6 weeks before progressing to full weight-bearing. At 1-year follow-up, the Knee Society Score (KSS) was 89 points (clinical) and 85 points (function), while the pain visual analog scale was 1 out of 10. The Western Ontario and McMaster Universities Osteoarthritis Index (WOMAC) total score was 15. Range of motion was from 0° extension to 110° flexion, with no evidence of mediolateral instability. At 9-year follow-up, the patient remained free of complications, including infection, aseptic loosening, or periprosthetic fracture; he reported a stable KSS of 82 points, with satisfaction rated as “very satisfied” (Figure 9b).

## Discussion

CNA is a progressive, non-infectious destructive arthropathy that develops in the setting of peripheral neuropathy, most commonly associated with long-standing diabetes mellitus (2). The condition is characterized by a paradoxical clinical presentation in which extensive joint destruction coexists with relatively preserved range of motion and disproportionately mild pain—a dissociation attributable to the loss of protective sensation (9). This unique feature frequently contributes to delayed or incorrect diagnosis, with potentially devastating consequences (6).

The diagnosis of CNA remains primarily clinical and radiological, as no pathognomonic histopathological features have been identified. In the early stages, joint swelling, effusion, and warmth may mimic infection or inflammatory arthritis, whereas advanced disease manifests as pathological fractures, joint dislocations, and severe deformity. The modified Eichenholtz staging system provides a valuable framework for classifying disease progression and guiding treatment decisions (10). *Stage 0* is characterized by acute inflammation without radiographic changes; *stage I* (development) shows fragmentation and bone destruction; *stage II* (coalescence) demonstrates absorption of debris and early sclerosis; and *stage III* (reconstruction) features consolidation and deformity (10).

The two cases presented herein illustrate the critical importance of early recognition and appropriate management of CNA involving the knee joint. In *case 1*, several diagnostic clues were overlooked. The patient sustained a comminuted tibial plateau fracture following minimal trauma—a fall while walking—yet remained able to bear weight and ambulate despite pain, an incongruity that should have raised suspicion for an underlying neuropathic process. In addition, intraoperative findings of severe bone fragmentation, osteolysis, and marked local osteoporosis were consistent with CNA but were not recognized. Furthermore, the postoperative analysis of synovial fluid, which was negative for microorganisms and crystals and showed a low white blood cell count, further ruled out pyogenic arthritis; however, these results were not fully recognized at the time as indicators of an underlying neuropathic process. This failure to consider CNA led to the performance of ORIF with bone grafting, a procedure with exceptionally high failure rates in this patient population.

A critical reflection on the intraoperative decision-making is warranted. Once the surgeon encountered a pale yellow effusion, extensive osteolysis, and multiple free-floating fragments in a patient with poorly controlled diabetes, the possibility of a neuropathic or infective process should have been formally entertained. At that point, a prudent course would have been to halt the procedure, obtain multiple deep tissue specimens for microbiology and histopathology, apply a spanning external fixator, and re-evaluate the diagnosis. However, the decision to proceed with internal fixation was likely influenced by the preoperative framing of the lesion as a traumatic non-union and the perceived urgency to stabilize a displaced intra-articular fracture. This case powerfully illustrates how cognitive anchoring bias can drive surgeons to continue a preoperative plan even when intraoperative findings contradict the original diagnosis. The subsequent failure of fixation underscores that achieving primary stability in Charcot neuroarthropathy is biologically impossible; the goal should instead be to control inflammation and protect the limb until definitive arthroplasty can be performed.

The poor outcome in *case 1* is not coincidental but rather reflects the fundamentally adverse biological and biomechanical environment for fracture healing and implant stability in CNA. Several interrelated factors contribute to this phenomenon. First, the neuropathic process disrupts normal bone metabolism and vascular autoregulation, resulting in progressive osteopenia and compromised bone stock, which diminishes screw purchase and impairs biological healing capacity (11). Second, persistent ligamentous laxity combined with loss of protective sensation precludes appropriate offloading of the surgical construct, leading to repetitive microtrauma and mechanical failure (12). Third, the biological milieu in CNA, characterized by abnormal cytokine activity, impaired vascularity, and chronic inflammation, creates a pronounced risk of non-union or delayed union (13). Collectively, these factors render conventional ORIF an unreliable treatment strategy for CNA-associated fractures, a principle increasingly recognized in the literature (14, 15).

In contrast, *case 2* exemplifies the favorable outcomes achievable with timely diagnosis and appropriate staged management. The diagnosis of CNA was established early (modified Eichenholtz stage I) based on the combination of minimal trauma, diabetic neuropathy, characteristic MRI findings including extensive bone marrow edema and fragmentation, and the classic clinical–radiographic dissociation. Initial conservative measures—protected weight-bearing, bracing, and bisphosphonate therapy—aimed to control the acute inflammatory phase and limit further bone destruction. Ultimately, when progressive joint destruction necessitated surgical intervention, the patient underwent TKA with stemmed components, a strategy specifically designed to address the unique challenges of CNA.

While TKA in patients with CNA has historically been associated with higher complication rates than in patients with primary osteoarthritis, contemporary evidence supports satisfactory long-term outcomes when specific technical principles are observed (16, 17). Critical considerations include meticulous ligament balancing to achieve coronal and sagittal stability; adequate bone resection with management of residual defects using cement, bone graft, or metallic augments; and routine use of stemmed components to enhance implant stability and offload stress from compromised bone (17, 18). The use of stemmed prostheses in this setting is particularly important, as it distributes forces beyond the affected metaphyseal region and mitigates the risk of loosening in osteopenic bone. The 9-year complication-free follow-up in *case 2* attests to the durability of this approach when properly executed.

While prior reviews have summarized the general management of Charcot knee, the present cases contribute specific new insights. First, they provide a direct head-to-head comparison between ORIF and staged TKA for the same anatomical site (tibial plateau) in diabetic CNA, an injury pattern for which comparative data are exceedingly scarce. Second, the 9-year complication-free follow-up after cemented stemmed TKA represents one of the longest reported successful outcomes in this population, demonstrating that durable fixation is achievable even in the neuropathic knee when CNA is recognized early and the acute inflammatory phase is allowed to subside before surgery. Third, the documented failure of ORIF despite an acceptable radiographic appearance at the time of surgery reinforces the concept that the hostile biological and biomechanical milieu of CNA—rather than technical error—is the primary driver of mechanical collapse. These findings strengthen the existing recommendation that conventional fracture fixation should be avoided in acute CNA of the knee and that arthroplasty, when performed with stemmed components and ligamentous constraint as needed, is a viable salvage procedure.

Several important lessons can be drawn from the present cases. First, CNA should be considered in any diabetic patient presenting with a fracture disproportionate to the mechanism of injury, particularly when pain appears relatively mild relative to the radiographic extent of disease. Second, intraoperative findings of severe fragmentation, osteolysis, and poor bone quality in the absence of infection or neoplasm should prompt reconsideration of the underlying diagnosis. Third, ORIF for CNA-associated fractures carries an unacceptably high failure rate in this population and should be reserved for highly selected patients with well-controlled neuropathy and a thorough understanding of the risks. Finally, when joint destruction is advanced, TKA with stemmed components represents a durable treatment option that can provide satisfactory long-term outcomes.

This study has several limitations. First, the sample size is very small (two cases), which precludes any statistical inference and limits generalizability. Second, a standardized diagnostic protocol for CNA was not applied, and some investigations (e.g., baseline neuropathy grading in case 1, serial inflammatory markers in case 2) were performed retrospectively from available records. Third, objective functional outcome scores and patient-reported outcome measures were not collected systematically at any follow-up points; the 9-year KSS for case 2 was obtained only at the final clinic visit. Fourth, the contrasting outcomes may be attributable in part to differences in baseline glycemic control and patient compliance rather than solely to diagnostic timing, although this does not diminish the core message. Finally, the retrospective nature of the report introduces inherent selection and recall bias. Despite these limitations, the vivid clinical contrast offers valuable cautions and guidance for surgeons facing similar scenarios.

## Data Availability

The raw data supporting the conclusions of this article will be made available by the authors, without undue reservation.
